# MicroRNA-567 dysregulation contributes to carcinogenesis of breast cancer, targeting tumor cell proliferation, and migration

**DOI:** 10.1007/s10549-016-4079-2

**Published:** 2016-12-20

**Authors:** Gloria Bertoli, Claudia Cava, Cecilia Diceglie, Cristina Martelli, Giampiero Rizzo, Francesca Piccotti, Luisa Ottobrini, Isabella Castiglioni

**Affiliations:** 1 Institute of Molecular Bioimaging and Physiology, National Research Council (IBFM-CNR), Via F.Cervi 93, 20090 Segrate-Milan, Milan, Italy; 2 Tecnomed Foundation, University of Milano-Bicocca, Monza, Italy; 3 Department of Pathophysiology and Transplantation, University of Milan, Milan, Italy; 4 Department of Medical Oncology, IRCCS Fondazione Maugeri, Pavia, Italy; 5 Laboratory of Experimental Oncology and Pharmacogenomics, Institutional Oncologic Bio-Bank “Bruno Boerci”, IRCCS Fondazione Salvatore Maugeri, Pavia, Italy

**Keywords:** MicroRNA/miRNA, Breast cancer, Prognosis, Biomarker, Proliferation

## Abstract

**Purpose:**

We demonstrated that *Hsa-miR-567* expression is significantly downregulated in poor prognosis breast cancer, compared to better prognosis breast cancer, having a role in the control of cell proliferation and migration by regulating *KPNA4 *gene.

**Methods and results:**

In this study, based on our previously published in silico results, we proved both in vitro (cell line studies) and ex vivo (clinical studies), that *Hsa*-*miR*-*567* expression is significantly downregulated in breast cancer with poor prognosis when compared to breast cancer with better prognosis. More intriguingly, we demonstrated that the ectopic expression of *Hsa*-*miR*-*567* in poor prognosis breast cancer cell line strongly inhibits in vitro cell proliferation and migration. Furthermore, we showed in vivo that breast cancer cells, stably expressing *Hsa*-*miR*-*567*, xenografted in mouse, reduce tumor growth ability. Consistently, we found that karyopherin 4 (*KPNA4*), predicted target gene of *Hsa*-*miR*-567 as identified by our in silico analysis, is upregulated in highly aggressive MDA-MB-231 breast cancer cell line and patient tissues with poor prognosis with respect to good prognosis.

**Conclusions:**

Our results suggest a potential role of *Hsa*-*miR*-*567* as a novel prognostic biomarker for BC and as regulator of *KPNA4*.

## Introduction

Breast cancer (BC) is one of the most common cancers worldwide and the most frequent women tumor (25% of all new cases diagnosed in 2015) [14, 25]. Although the current histological and biological indexes (i.e., tubule formation, nuclear polymorphism, mitotic count,…) [2, 10] have shown association with clinical outcome of BC patients, these indexes are limited in their ability to make prognosis for all BC patients.

With the advent of microarray and the sequencing of the human genome, transcriptomic analysis has significantly improved the knowledge of the biological mechanisms leading to malignant transformation of BC, providing better molecular ‘portrait’ of BC and allowing the identification of new BC prognostic and predictive tools.

Several gene profiles have been proposed for the classification of BC [i.e., the 105-gene profile by Perou [24], 70-gene profile in Mammaprint by van ‘t Veer [29], 21-gene profile in Oncotype by Paik [22], 97 gene profile by Sotiriou [26], 18 gene profile, and 6 gene profile by Ivshina [13]]. Notwithstanding these gene profiles have few genes in common, they have consistently shown that BC patients can be classified into two groups with different prognosis, namely grade 1 (G1)-like and grade 3 (G3)-like groups, representing the two different prognostic groups defined by histological analysis as G1 and G3 [13].

MicroRNAs (miRNAs or miRs) are small, highly conserved, non-coding RNAs that regulate gene expression of target mRNAs. They are widely involved in several physiologic processes, but they are also responsible for pathological conditions, such as carcinogenesis and cancer development [11]. miRNA profiles obtained by ‘omics’-based technology can classify human cancers even better than mRNA profile [19, 27]. Moreover, miRNAs are emerging as easy accessible cancer disease biomarkers, as they have been found stably present in human biofluids (i.e., blood, saliva, urine…) [3, 4]. These features make miRNAs interesting, non-invasive, and cost affordable biomarkers for clinical diagnosis, prognosis, and therapy of BC.

By combining gene expression profile, copy number alterations, and miRNA expression profiles as available from published datasets of BC human tissue samples, we have recently identified in silico, a 4-miRNA signature (*Hsa*-*miR*-*567, Hsa*-*miR*-*139*-*5p, Hsa*-*miR*-*320d, Hsa*-*Let*-*7c*) and a 4-target mRNA signature (*KPNA4*, *H2AFV*, *FOXM1*, *DDX19A*) that are able to accurately classify BC patients into G1-like and G3-like groups, thus improving BC grade definition and confirming the existence of only two prognostic groups [5]. Our signature was the first one, to our knowledge, consisting of miRNAs with these prognostic properties in BC. In particular, it was the first time that *Hsa*-*miR*-*567* (*miR*-*567*) was found significantly downregulated in BC.

Following our preliminary in silico results, the present study aims at elucidating the role of *miR*-*567* for the prognosis of BC and as regulator of karyopherin 4 (*KPNA4)*, and to investigate the effects caused by a dysregulation of *miR*-*567.* Specific aims of our study are to assess the following, by experimental studies: (1) if the expression level of *miR*-*567* is significantly downregulated in BC with poor prognosis when compared to BC with good prognosis (and, consistently, if the expression level of *KPNA4* is significantly upregulated), and (2) *miR*-*567* modulation effects on BC tumor cell proliferation and migration. To reinforce the evidences of a crucial role in BC of *KPNA4* and *miR*-*567,* we implemented a classification algorithm and evaluated the performances of proposed biomarkers in the classification of G1 and G3 BC and in the re-classification of G2, using an independently collected Gene Expression Omnibus (GEO) dataset.

## Materials and methods

### BC cell line

For in vitro studies, we used two human BC epithelial cell lines: G1-like MCF7 and G3-like MDA-MB-231 cells [12] (ICLC-Biologic Bank and Cell Factory, Italy). We have chosen MCF7 and MDA-MB-231 cell lines for their ability to form moderately well-differentiated infiltrating ductal carcinomas (grade I) and poorly differentiated adenocarcinoma (grade III), respectively, when xenografted in mouse [https://www.lgcstandards-atcc.org/Products/All/HTB-26.aspx#characteristics] [12, 31]. Following the manufacturer’s recommendation, we maintained the cell lines within a humidified atmosphere containing 5% CO_2_ at 37 °C in DMEM cell culture medium (Gibco, Life Technologies), with 10% fetal bovine serum (FBS) (Lonza, Euroclone). Dulbecco Phosphate-Buffered Saline (D-PBS), trypsin, and all the media additives (penicillin, streptomycin, d-glutamate, non-essential amino acids) were obtained by Lonza (Euroclone).

### BC human tissue samples

We used 13 G1 and 13 G3 snap-frozen human BC tissues, diagnosed histopathologically at “Salvatore Maugeri” Foundation, at the Histopathology Service, from 2009 to 2015. G1 and G3 grade definition was established according to the Scarff-Bloom-Richardson (SBR) grading system [1].Tissue samples were immediately stored as frozen aliquots in the “Bruno Boerci” Institutional Oncologic Bio-bank after surgery until further use, according to the guidelines of European Bio-banking and Biomolecular Resource.

### BC animals and animal tissue samples

Fourteen adult female nude mice (athymic nude-Foxn1 Nu/Nu), 7–8 weeks old, were used for the in vivo experiment, and xenografted to model BC.

During in vivo study, mice were maintained on a 12-h light–dark cycle in cages of five animals with water and food ad libitum.

For ex vivo studies, at the end of the experiments, mice were sacrificed by cervical dislocation after sedation and five explanted tumors, from five mice were immediately stored in liquid nitrogen for further use.

### RNA isolation and reverse transcription (in vitro and ex vivo studies)

Total RNA was isolated using TRIzol reagent (Life Technologies) following the manufacturer’s recommendations.

Two micrograms of total RNA were reverse transcribed using miRCURY LNA Universal RT microRNA PCR (Exiqon, Euroclone) to obtain poly-A-tailed miRNA cDNA. U6 spike-in RNA was added in the reaction as an internal control for the reverse transcription and real-time PCR (RT-PCR) amplification.

Two micrograms of total RNA, treated with DNAse I (Euroclone, Italy), were reverse transcribed using oligo dT primers in combination with SuperScript II Reverse Transcriptase (Life Technologies) in order to obtain total cDNA for gene expression analysis, following the manufacturer’s protocol.

### RT-PCR for analysis of *miR-567 *and *KPNA4* expression levels

*miR*-*567* and *KPNA4* expression levels were analyzed on BC cell lines and human samples. RT-PCR was performed using SybrSelect Master Mix (Applied Biosystem, Life Technologies), in an Eco RT-PCR (Illumina).

Primer sequences for *miR*-*567* expression analysis have been purchased from Exiqon (Euroclone, Italy) (Accession No. MI0003573). The level of expression of *miR*-*567* was normalized on the level of U6 control gene.

Primer sequences for gene expression analysis are: for *KPNA4* (ID3840) Fw: 5′- CAGGAGATTCTTCCAGCCCTTTGTGT-3′, Rw: 5′- ATTACCATCTGTATTTGTTCATTGCCAGCATC-3′. For ribosomal protein S14 (*RPS14)* (ID6208) Fw: 5′- GGCAGACCGAGATGAATCCTCA-3′, Rw: 5′- CAGGTCCAGGGGTCTTGGTCC-3′. *RPS14* was used as internal control for RT-PCR quantification.

The relative expression of *miR*-*567* and *KPNA4* was calculated for both G3 and G1 cell lines and human samples with the $2^{{ (- \Delta \Delta {\text{C}}_{\text{T}} )}}$ method [17]. Experiments were performed three times in triplicate (*n* = 9). A *t* test was calculated among G3 and G1 cell lines and human samples.

### *miR-567* stable transfectant generation in G3-like cell line: *miR-567* and *KPNA4* analyses

MDA-MB-231 cells stably overexpressing *miR*-*567* (MDA-miR) or scramble sequence (MDA-Scr) (for the scramble sequence see [18]) were obtained by seeding 50,000 cells in 24-well plate (Euroclone) and transfecting them with 500 ng of pCMV-miR567-GFP (Origene, Nockville, MD) or 100 nM scramble, respectively, in the presence of Lipofectamine 2000 reagent (Invitrogen), following the manufacturer’s suggestions. After 48 h, the medium was changed, and G418/Neomycin (Euroclone) 500 ug/ml was added. The whole population was maintained for 3 weeks in selection (3 passages/week) to obtain a stable transfectant cell line.

After 3 weeks, to assess the efficiency of the stable cell transfection, we evaluated the expression levels of *miR*-*567* and *KPNA4* in the whole population of MDA-miR versus MDA-Scr by RT-PCR, using the same protocol described above.

### *miR-567 *stable transfectant generation in G3 cell line: Kpna4 protein analysis

Total lysates were obtained by lysing 1 × 10^6^ of MDA-ctr cells and MDA-miR cells 30 min in RIPA lysis buffer (50 mM Tris, pH 7.5, 150 mM NaCl, 1% NP-40, 2% SDS, 0.15% deoxycholic acid, 1 mM EDTA, pH 8. 0) containing complete protease inhibitor (Roche, Monza, Italy), leupeptin and aprotinin inhibitors (Sigma Aldrich). Lysates were spun at 12,000×*g* for 30 min and the supernatants collected. Estimation of protein concentration was performed with 660 nm Protein assay (Pierce, Thermo Scientific, France) on a Fluostar Omega plate reader (BMG Labtech, Germany).

Twenty micrograms of total proteins were loaded on 8.5, 10, or 12% SDS–PAGE and run for 1.5 h at 30 mA/gel. Proteins were transferred onto nitrocellulose membrane (Sigma Aldrich) at 250 mA for 4 h. Membranes were blocked in TBS-0.1% Tween-20 containing 5% skim milk powder (Euroclone, Italy), and incubated with primary antibodies: rabbit polyclonal anti-beta Actin (OriGene, MD, USA), anti-Histone H3 (Origene, MD, USA), or anti-Tubulin (sc-5286, Santa Cruz Biotechnologies) used 1:000; rabbit polyclonal anti-Kpna4 (Thermo Fisher Scientific, IL, USA) diluted 1:750. After incubation with goat anti-rabbit HRP secondary antibody (sc-2004, Santa Cruz Biotechnologies) or goat anti-mouse HRP (sc-2005, Santa Cruz Biotechnologies) at 1:5000, membranes were developed using the SuperSignal West Pico Chemiluminescent Substrate (ThermoScientific, France) on Amersham Hyperfilm MP (Ge Helthcare, Italy).

To assess the stable transfection, we evaluated the expression levels of Kpna4 protein in MDA-ctr and MDA-miR by Western Blot analysis, normalizing the Kpna4 protein to histone H3. The blots of both proteins were quantified by ImageJ software (http://imagej.nih.gov/ij/). Experiments were performed two times in triplicate (*n* = 6). A *t* test was calculated among MDA-miR and MDA-ctr.

### Luciferase assay

To assess if *KPNA4* is a direct target of *miR*-*567*, pMiRtarget vector, containing the 3′UTR of human *KPNA4* sequence (Origene, Nashville), was transfected with lipofectamine 3000 (Invitrogen) in MDA-MB-231 cells, according to the manufacturer’s suggestion, in combination with 100 nM mimic *miR*-*567* or 100 nM scramble miR sequence. After 24 h, Luciferase activity was measured in GloMax-Multi Detection System (Promega, Madison, WI, USA) and normalized on total protein content. Protein quantification has been performed with Bradford method (Promega).

### In vitro study of tumor cell proliferation

Tumor cell viability was assessed by following the growth of 50,000 MDA-ctr cells or MDA-miR, seeded in 24-multiwell plate. At time 0, 24, 48, 72 h from seeding, the cells were gently washed with PBS, trypsinized and count with Trypan Blue.

A graphic representation of the results was obtained by plotting the number of the total cells at each time point. Experiments were performed three times in triplicate (*n* = 9). A *t* test was calculated among MDA-miR and MDA-ctr.

### In vitro study of tumor cell migration

To study directional cell migration, we performed a wound-healing study. The MDA-ctr and MDA-miR cells were seeded at confluence (about 1,50,000/well) in 24-well plate (Euroclone). After 6 h, necessary for adhesion, a scratch was performed in the middle of each well [16]. After washing the cells, new medium was added, and pictures of each well were taken every 24 h. The effect on cell migration was quantified by ImageJ software. Experiments were performed three times in triplicate (*n* = 9). A *t* test was calculated among MDA-miR and MDA-ctr.

### In vivo study of tumor growth

To engineer cells, a pCCL.PGK.luciferase.WPRE (PLW) lentivector was in vitro used (kindly provided by Dr. S. Rivella). The construct permitted the expression of luciferase reporter gene under control of phosphoglycerate kinase 1 (PGK) promoter.

PLW lentiviral particles were produced by transient transfection into 293 T cells using calcium phosphate precipitation, as described elsewhere [6]. The transfection medium (IMDM, Euroclone, plus 10% FBS, Cellgrow) was replaced 16 h later. The conditioned medium, containing the viral particles, was collected 24 and 48 h after the medium replacement, filtered through cellulose acetate filter 0.2 μm, and frozen at −80 °C until further use. The vector titer was determined by serial dilution on HeLa cells through Real-Time PCR.

MDA-ctr or MDA-miR cells were plated and infected with PLW particles with a MOI of five in the presence of Polybrene (8 ug/ml), generating MDA-ctr + Luc (MDA-Luc) or MDA-miR + Luc (MDA-miR-Luc). Cells were washed 16 h later in order to remove all viral particles.

Five millions of cells either from MDA-Luc or MDA-miR-Luc were resuspended in 40 μl of sterile PBS and injected orthotopically in the fourth mammary gland of the animals. Briefly, mice were anesthetized with Zoletil + Xilor 2% (40 and 8 mg/kg, respectively) and a 4-mm incision was performed on the skin, in the proximity of the mammary gland. The needle of the syringe was inserted to the mammary gland, and cells were delivered. Following, skin incision was sutured with a 6/0 silk suture thread and mice were followed until complete recovery.

To analyze tumor growth, bioluminescence imaging was performed at different time points. After anesthetization, the 14 mice were injected with 50 mg/kg of luciferin (Beetle Luciferin Potassium Salt, Promega), and after biodistribution, bioluminescence signal was acquired.

All acquisitions were carried out with IVIS SPECTRUM/CT system (PerkinElmer). Images were analyzed and normalized, using the Living Image Software. Data were expressed as average radiance (photons/seconds/cm^2^/steradian). The scale and the average radiance used for the images took into account the difference in luciferase activity for the two cell lines.

Dimension of all 14 tumors was measured by caliper on animals at different time points. The tumor volumes were calculated by multiplying the three dimensions of the tumor measured by caliper.

In order to assess if the upregulation of *miR567* was maintained during the in vivo study, five explanted tumors from five xenografted animals, generated by MDA-Luc or MDA-miR-Luc, were collected, weighted, and analyzed for RNA, mRNA, and protein content by RT-PCR or Western blot.

### Bioinformatic classification approach

In silico validation analysis using *KPNA4* and *miR*-*567* was performed from an independent dataset of GEO database: GSE22220. This dataset contains mRNA and miRNA expression profiles from the same patients: 41 G1 BC, 81 G2 BC, and 61 G3 BC samples.

In order to evaluate the performances of the two proposed biomarkers, we developed a Support Vector Machine (SVM) classification model using the R-package: e1071 [20]. AUC was estimated by cross-validation method (k-fold cross-validation, *k* = 10).

We optimized inference AUC over a space of given SVM feasible learning parameters: ${\text{cost}} = 10^{( - 1:2)}$ , gamma = c(0.2,1,2); kernel type = RADIAL (see e1071 documentation at [20] (https://CRAN.R-project.org/package=e1071]). This approach allowed us to find the best SVM learning parameters for each data type over the same space of values.

We implemented a Monte Carlo Cross-Validation method. It randomly selected some fractions of GEO data (60% of original dataset) to form the training set, and then assigned the rest of the points to the testing set (40% of original dataset). This process was then repeated multiple times (ten bootstraps), generating randomly new training and test partitions each time. For each bootstrap, we obtained an AUC value.

In order to avoid problems of unbalanced classes, we randomly selected classes with the same number of BC sample in both the training and testing dataset for each evaluation.

The classifier was tested for the following reasons: (1) to distinguish histological G1 and G3 BC patients using *miR*-*567* and *KPNA4* expression profiles and (2) to re-classify G2 using *miR*-*567* and *KPNA4*. For this purpose, the machine learning algorithm based on SVM was used to classify G2 BC patients in G1 or G3 class. The results of testing G2 samples were classified as G1 or G3 (namely G1* and G3*). These classes G1* and G3* were used to train SVM again and to test to the different G1 and G3 BC datasets.

## Results

### *miR-567 *is significantly downregulated and its target *KPNA4 *is significantly upregulated in G3 versus G1 BC cell lines

**Fig. 1:**
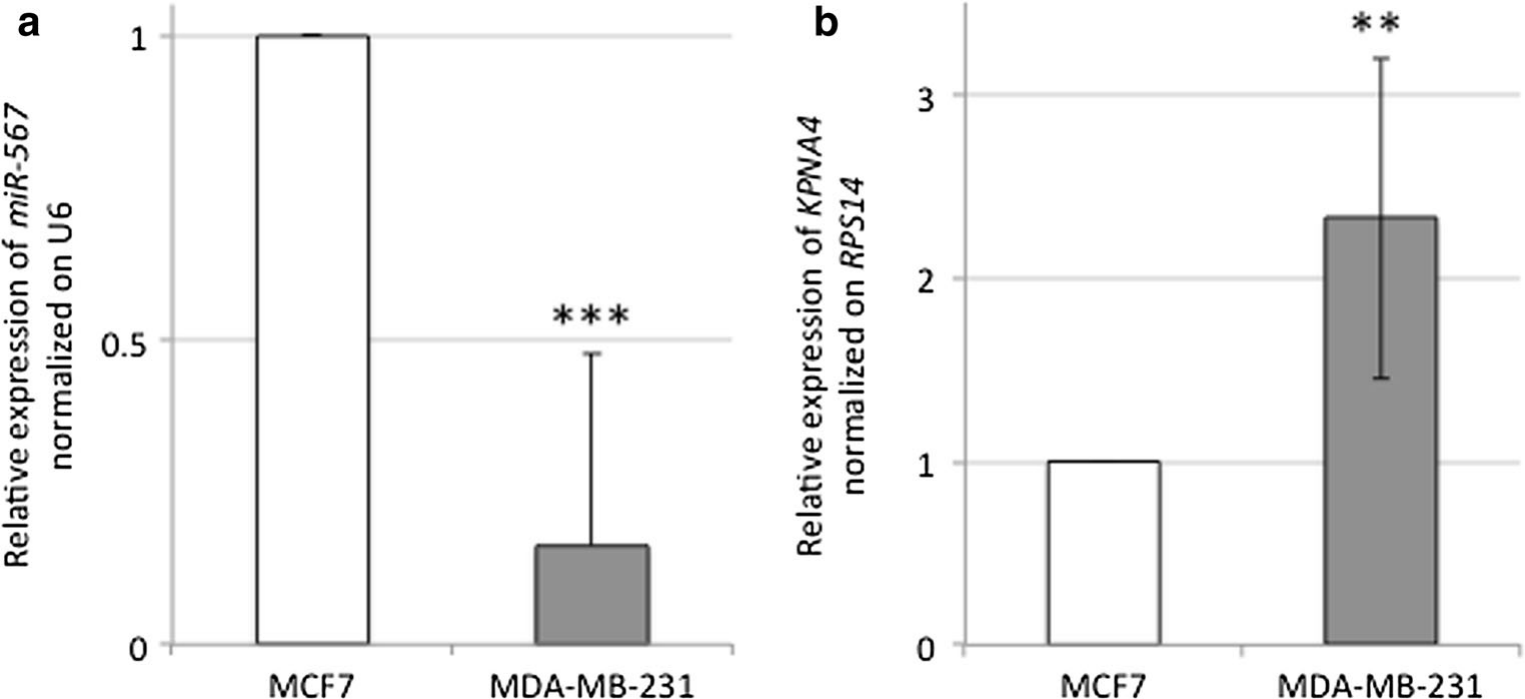
**a** RT-PCR relative expression of *miR*-*567* normalized on U6 housekeeping gene, in MCF7 (in *white*) and MDA-MB-231 (in *gray*) cell line, respectively. All data are presented as mean ± SD of experiments for each bar (***t test *p* value = 4.5 × 10^−5^, *n* = 9). **b** RT-PCR relative expression of *KPNA4* gene normalized on *RPS14* housekeeping gene, in MCF7 (in *white*) and MDA-MB-231 (in *gray*) cell line respectively. All data are presented as mean ± SD of experiments for each bar (**t test *p* value = 0.0048, *n* = 9)

To validate that the expression level of *miR*-*567* is downregulated and *KPNA4* mRNA is upregulated in G3 versus G1 BC, we performed a RT-PCR in MDA-MB-231 and MCF7 cell lines. As shown in Fig. 1a, *miR*-*567* expression in MDA-MB-231 is significantly downregulated compared to MCF7 cell line. As shown in Fig. 1b, the relative expression of *KPNA4* mRNA is significantly higher in MDA-MB-231 versus MCF7 cell line.

### *miR-567* is significantly downregulated and *KPNA4 *is significantly upregulated in G3-like versus G1-like BC human tissues

**Fig. 2:**
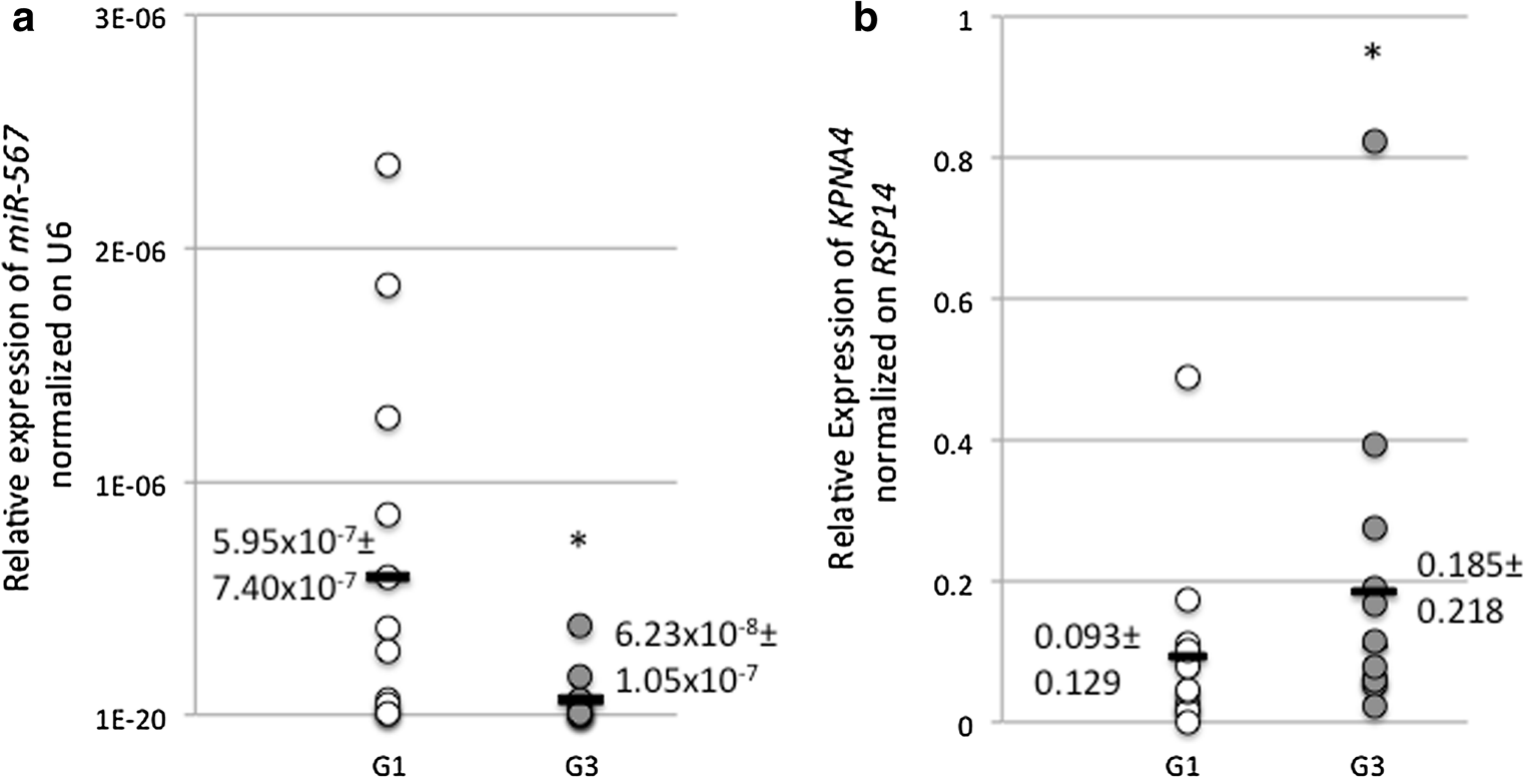
**a** Relative expression of *miR*-*567* normalized on U6 in 13 G1 (in *white*) versus 13 G3 (in *gray*) BC. All data are presented as mean ± SD of experiments for each point. *Black bars* represent the average of each group. (*t test *p* value = 0.0223, *n* = 13). **b** Relative expression of *KPNA4* normalized on *RPS14* in 13 G1 (in *white*) versus 13 G3 (in *gray*) BC. All data are presented as mean ± SD of experiments for each point. *Black bars* represent the average of each group (*t test *p* value = 0.047, *n* = 13)

To validate that the expression level of *miR*-*567* is downregulated in G3 versus G1 BC human tissue samples and that expression level of *KPNA4* is lower in G1 samples versus G3 samples, we performed a RT-PCR in 13 G3 versus 13 G1 samples. In Fig. 2a, we showed that the expression level of *miR*-*567* is lower in G3 human tissue samples versus G1 samples, with an average of 5.95 × 10^−7^ ± 7.40 × 10^−7^ in G1 and of 6.23 × 10^−7^ ± 1.05 × 10^−7^ in G3 samples, respectively. In Fig. 2b, we showed that the expression level of *KPNA4* is lower in G1 samples versus G3 samples, with an average of 0.185 ± 0.218 in G3 and of 0.093 ± 0.129 in G1 samples, respectively.

### *miR-567* modulation has effects on BC cell proliferation and migration

**Fig. 3:**
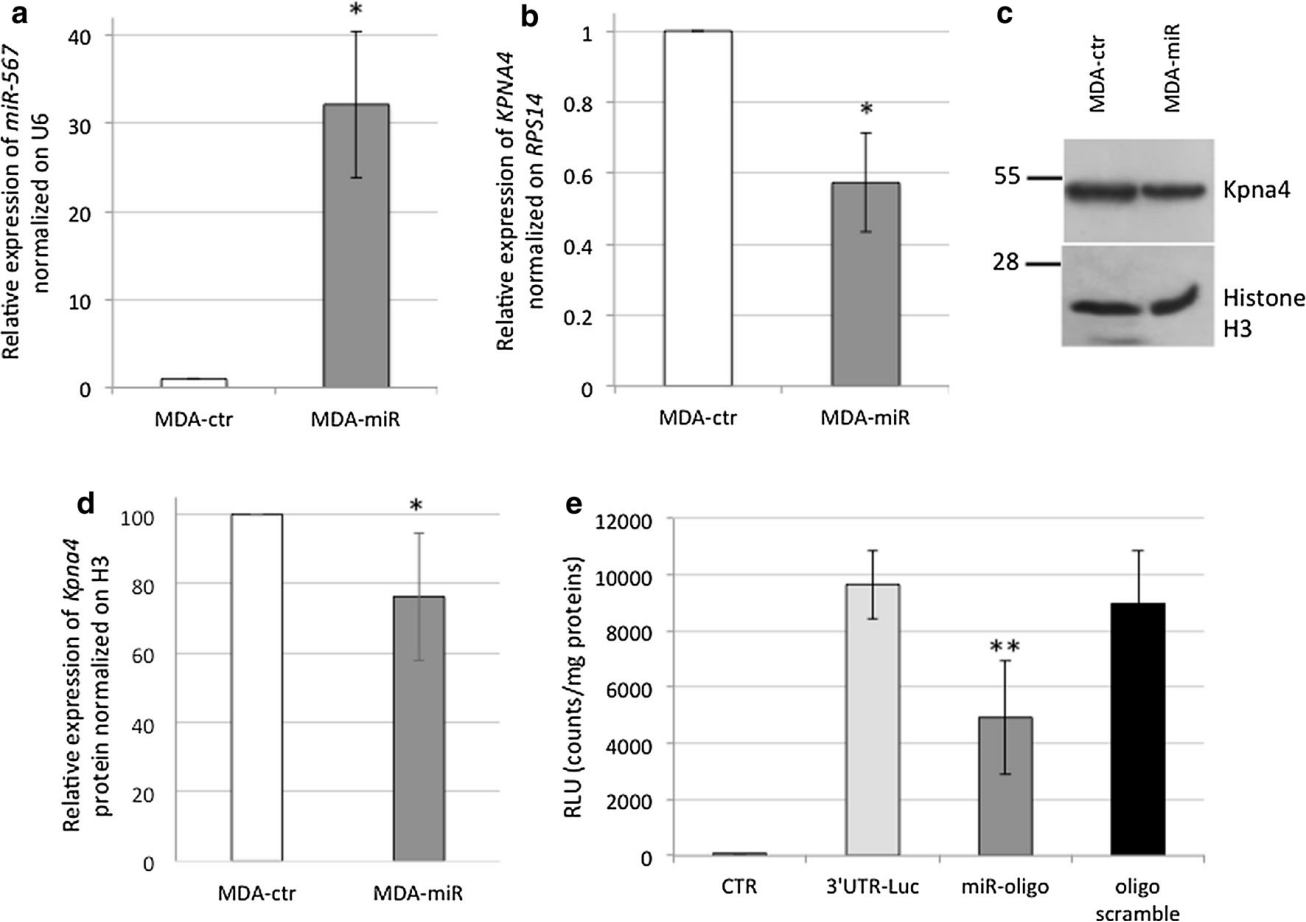
**a** Relative expression of *miR*-*567* normalized on *U6* in MDA-ctr cells (in *white*) versus MDA-miR (in *gray*). All data are presented as mean ± SD of experiments for each bar (*t test *p* value = 0.02, *n* = 9). **b** Relative expression of *KPNA4* normalized on RPS14 in MDA-ctr cells (in white) versus MDA-miR (in *gray*) cell lines, respectively. All data are presented as mean ± SD of experiments for each bar (*t test *p* value = 0.012, *n* = 9). **c** Western Blot of KPNA4 protein and Histone H3, used as normalizing protein. **d** Western Blot quantification of KPNA4 protein normalized on Histone H3 in MDA-ctr (in *white*) and MDA-miR (in *gray*) cells. All data are presented by mean ± SD of experiments for each bar (*t test *p* value = 0.05, *n* = 6). **e** Luciferase assay results are depicted in the graph. The relative luminescence activity (RLU) has been normalized on total protein content for each sample. CTR: untreated MDA-MB-231; 3′UTR: MDA-MB-231 transfected with pMirTarget vector containing 3′UTR of hKPNA4 sequence; 3′UTR + miRNA: MDA-MB-231 transfected with pMirTarget vector containing 3′UTR of hKPNA4 sequence, in combination with 100 nM mimic *miR*-*567* oligonucleotide; 3′UTR + scramble: MDA-MB-231 transfected with pMirTarget vector containing 3′UTR of hKPNA4 sequence, in combination with 100 nM scramble oligonucleotide. All data are presented as mean ± SD of experiments for each point (**t test *p* value = 0.0013, *n* = 3)

Since *miR*-*567* is downregulated in poor prognosis G3 cells, to evaluate the effect of *miR*-*567* upregulation on cell behavior, we generated a stable transfectant, overexpressing *miR*-*567* in MDA-MB-231 cell line. Figure 3a shows that the vector transfection significantly increased *miR*-*567* expression up to 32.15 ± 8.32 fold in MDA-miR versus MDA-ctr cells.

To evaluate the effect of *miR*-*567* overexpression on *KPNA4* target gene levels, we analyzed both the mRNA levels and the protein levels. As shown in Fig. 3b, *miR*-*567* stable upregulation decreased *KPNA4* mRNA expression to 0.57 ± 0.13 fold in MDA-miR versus MDA-ctr cells by RT-PCR analysis.

Western blot analysis revealed that Kpna4 protein is also reduced in MDA-miR compared to MDA-ctr cells (Fig. 3c) to 76.34 ± 18.48 (ImageJ quantification, Fig. 3d). Luciferase assay confirmed that *KPNA4* gene is a direct target of *miR*-*567* (Fig. 3e).

### Upregulation of *miR*-*567 *decreases tumor cell proliferation

**Fig. 4:**
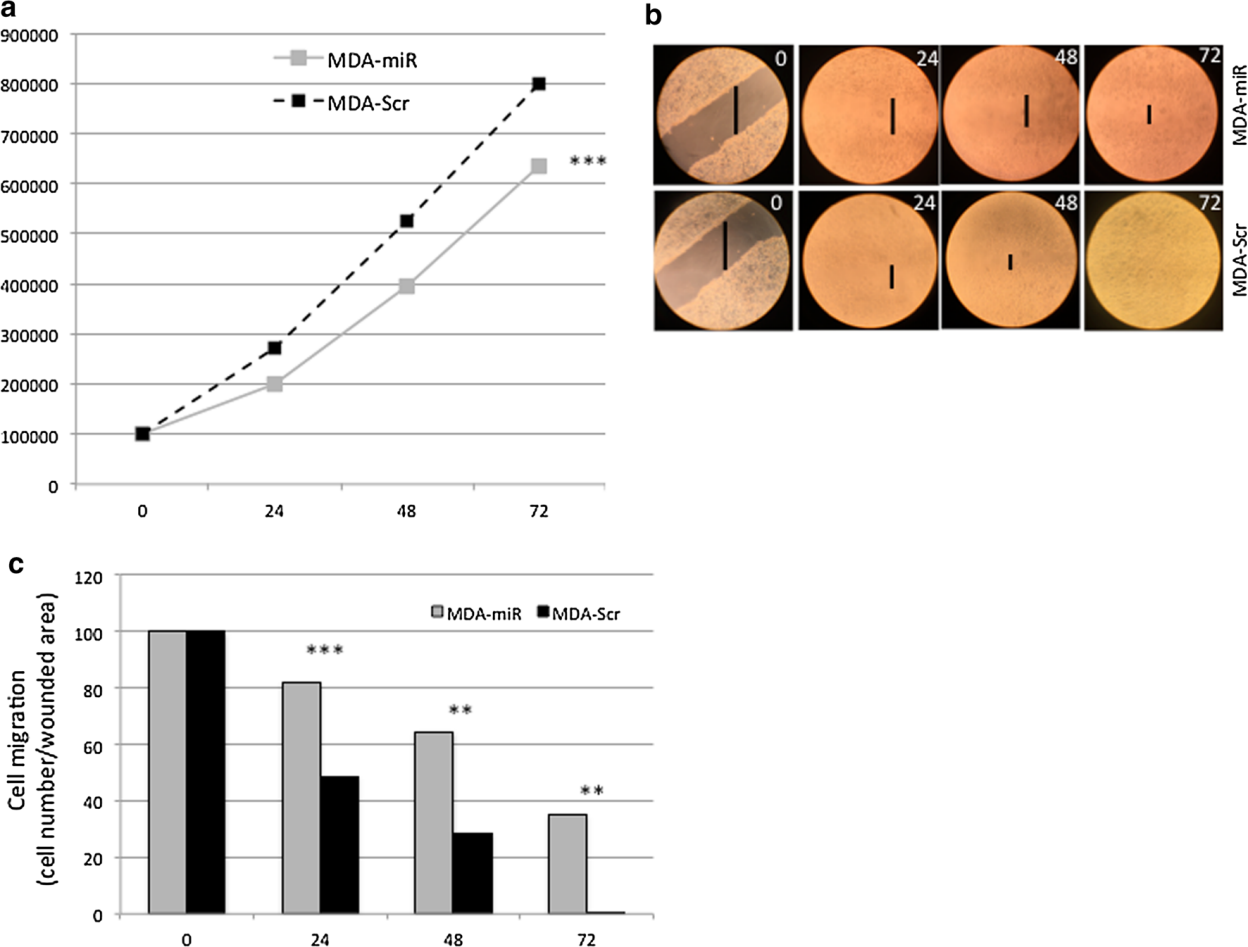
**a** Growth curve of MDA-Scr (*dashed black line*) and MDA-miR (*solid gray line*) cell lines. All data are presented as mean ± SD of experiments for each point at each time point (***t test *p* value = 1.28 × 10^−7^, *n* = 27). **b** Behavior of MDA-Scr and MDA-miR cells in wound-healing test. **c** ImageJ quantification of the effect of the *miR*-*567* overexpression in MDA-miR compared to MDA-Scr cells, on wound-healing assay. All data are presented as mean ± SD of experiments for each bar at each time point (**p value < 0.01, ***t test *p* value < 0.001, *n* = 3, respectively)

MDA-miR and the parental MDA-ctr cell lines were tested in growth curve assay (Fig. 4). The overexpression of *miR*-*567* decreased the proliferation of MDA-miR in respect to MDA-ctr cells (Fig. 4a). The percentage of apoptotic cells was not affected by the overexpression of *miR*-*567* (data not shown).

### Upregulation of *miR-567* decreases cell migration

To test the influence of *miR*-*567* overexpression on ability migration of BC cells, we performed wound-healing assay. MDA-miR showed a lower ability to heal the wound in the MDA-miR cell culture well compared to MDA-Scr cells (Fig. 4b). The difference in the wound area between the two cell lines, quantified by ImageJ, was statistically significant at time 24, 48, and 72 h (Fig. 4c).

### Upregulation of *miR-567 *decreases in vivo tumor growth

To test if *miR*-*567* affects the proliferation of BC tumor cells in vivo, we analyzed the behavior of MDA-miR and MDA-ctr implanted in mouse mammary gland. To follow the growth of the tumors, both MDA-miR and MDA-ctr cell lines were engineered in vitro by infection with the PLW construct, producing a detectable signal from luciferase activity. The efficiency of infection was about 90% (data not shown) and the MDA-miR cells showed a higher level of signal compared to MDA-ctr cells (ratio of MDA-miR/MDA-ctr = 5.7 fold)—this difference is only due to the differential amount of vector copies integrated in the two cell models (2.125:1 in MDA-miR versus MDA-ctr, respectively) and not to a difference in cell behavior.

**Fig. 5:**
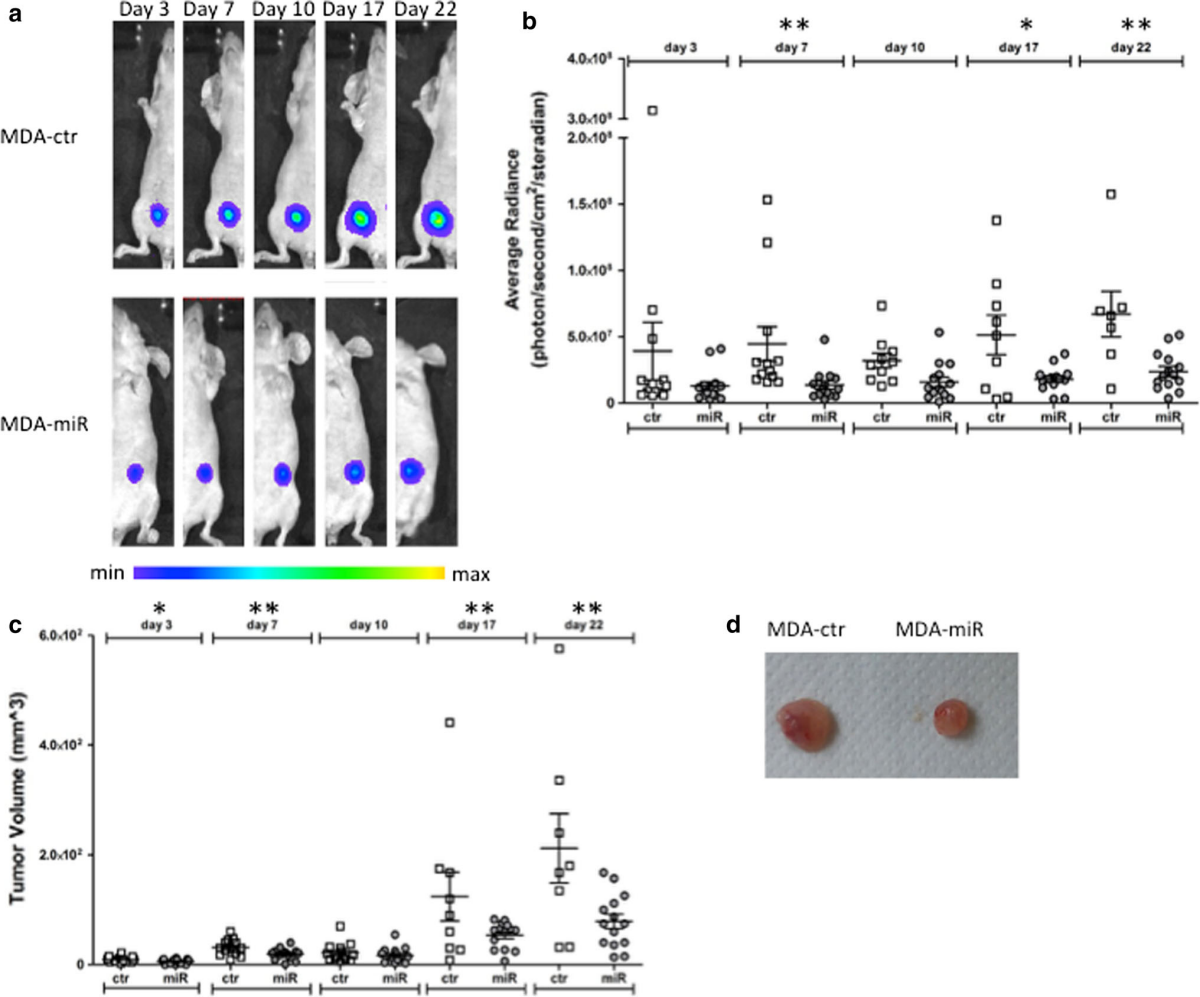
**a** Bioluminescence imaging of a representative mouse injected with MDA-ctr (control) cells or with MDA-miR cells. All the images are scaled with the same color bar. **b** Graphical representation of average of Luciferase activity acquired for each of the tumor formed by MDA-ctr (ctr) and MDA-miR (miR) cell lines, respectively, at different time points (day 3, 7, 10, 17, 22). Data are presented as average radiance (photons/s/cm^2^/steradian). All the bars are mean ± SEM of experiments for each cell line at each time point (**t test *p* value day 7 = 0.008; *t test *p* value day 17 = 0.015;** *t* test *p* value day 22 = 0.007, *n* = 14). **c** Graphical representation of average of volume for each of the tumor formed by MDA-ctr (ctr) and MDA-miR (miR) cell lines, respectively, at each time point (day 3 7, 10, 17, 22). All the bars are mean ± SEM of experiments for each cell line at each time point (*t test *p* value day 3 = 0.025; **t test *p* value day 7 = 0.010; **t test *p* value day 17 = 0.006; **t test *p* value day 22 = 0.002, *n* = 14). **d** Representative image of the difference in the volume of explanted tumors, generated by MDA-ctr (*left*) and MDA-miR (*right*) cells, respectively

As shown in Fig. 5a, a different tumor growing rate was observed by optical imaging: the overexpression of *miR*-*567* stable construct caused a delay in the growth of the tumors if compared to the growth of MDA-ctr generated tumors (image scale took into account the difference in luciferase constitutive activity of the two cell lines).

The quantification of bioluminescent signal by ROIs showed a very different rate of tumor growth, as demonstrated by the significant *t* test (Fig. 5b).

This result was confirmed by evaluation of tumor volumes, as recorded by caliper at each time point of signal acquisition, and of tumor weight at the end of experiment, showing a smaller tumor (both in volume and weight) formed by MDA-miR compared to MDA-ctr cells (Fig. 5c–d, respectively).

**Fig. 6:**
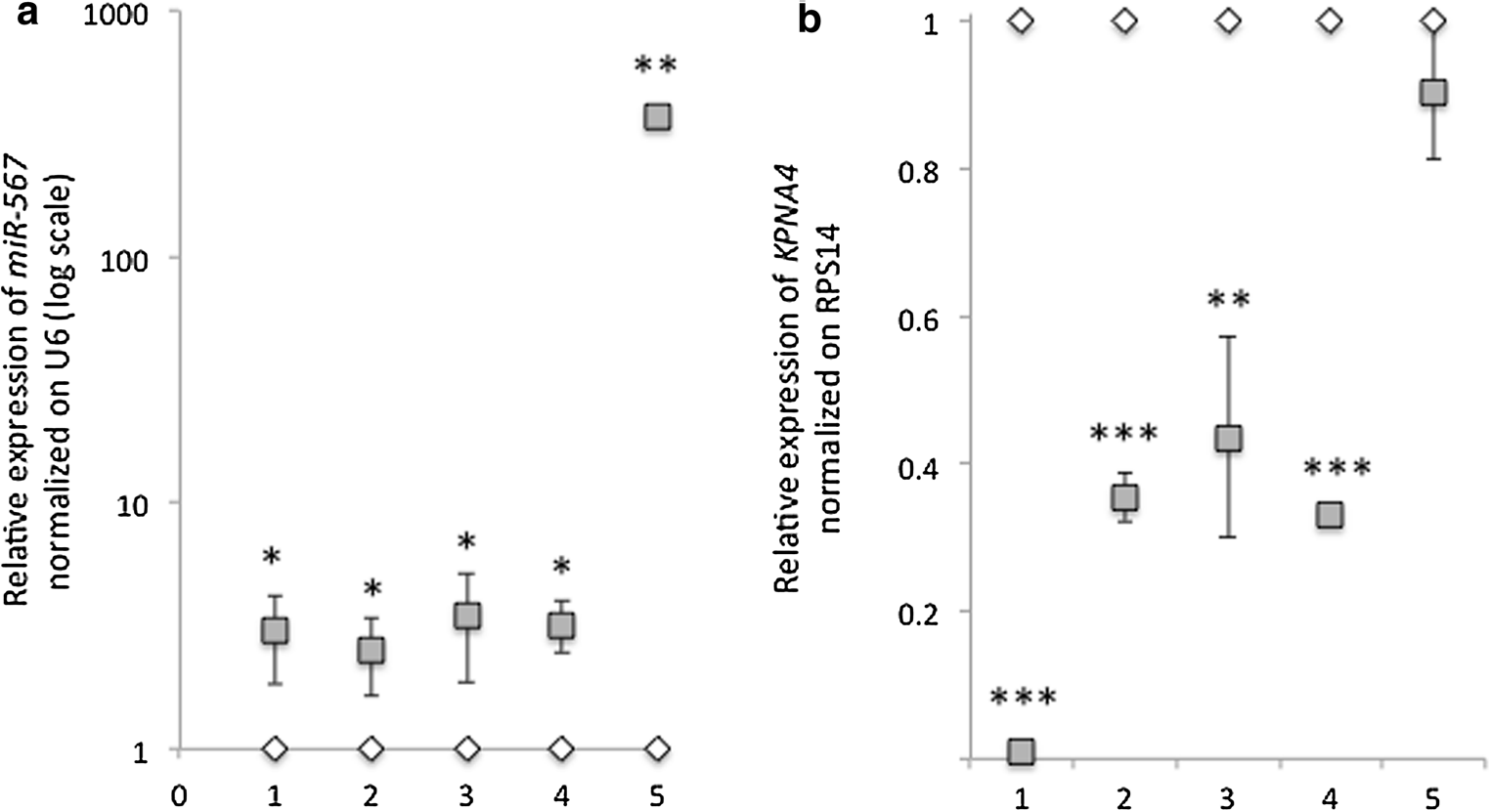
**a** Relative expression (log scale) of *miR*-*567* normalized on U6 on five explanted tumors formed by MDA-miR + luc (in *gray*) or MDA-ctr + luc (in *white*) cells, respectively. All data are presented as mean ± SD of experiments for each tumor (1–5) (*t* test *p value < 0.05; **p value < 0.01, *n* = 9). b) Relative expression of *KPNA4* normalized on *RPS14* on five explanted tumors (1–5) formed by MDA-miR + luc (in *gray*) or MDA-ctr + luc (in *white*) cells, respectively. All data are presented as mean ± SD of experiments for each tumor (*t* test **p value < 0.05; ***p value < 0.001, *n* = 9)

On the explanted tumors, as expected, *miR*-*567* expression was maintained upregulated in MDA-miR compared to MDA-ctr cells (fold change expression was in mouse 1: 3.03 ± 1.20; in mouse 2 = 2.52 ± 0.87; in mouse 3 = 3.51 ± 1.65; in mouse 4 = 3.22 ± 0.77, respectively) (Fig. 6a). One sample (mouse 5) showed an outlier expression of *miR*-*567*, with a relative expression of 366.67 ± 2.34 fold of increase.

Consistently, on the explanted tumors, *KPNA4* expression was found downregulated in MDA-miR compared to MDA-ctr cells (fold change expression of *KPNA4* was in mouse 1: 0.01 ± 0.01; in mouse 2 = 0.35 ± 0.03; in mouse 3 = 0.44 ± 0.14; in mouse 4 = 0.33 ± 0.01; in mouse 5 = 0.90 ± 0.09, respectively) (Fig. 6b).

### Bioinformatic approach to evaluate the performance of *miR-567* and *KPNA4* in G1–G3 classification

**Fig. 7:**
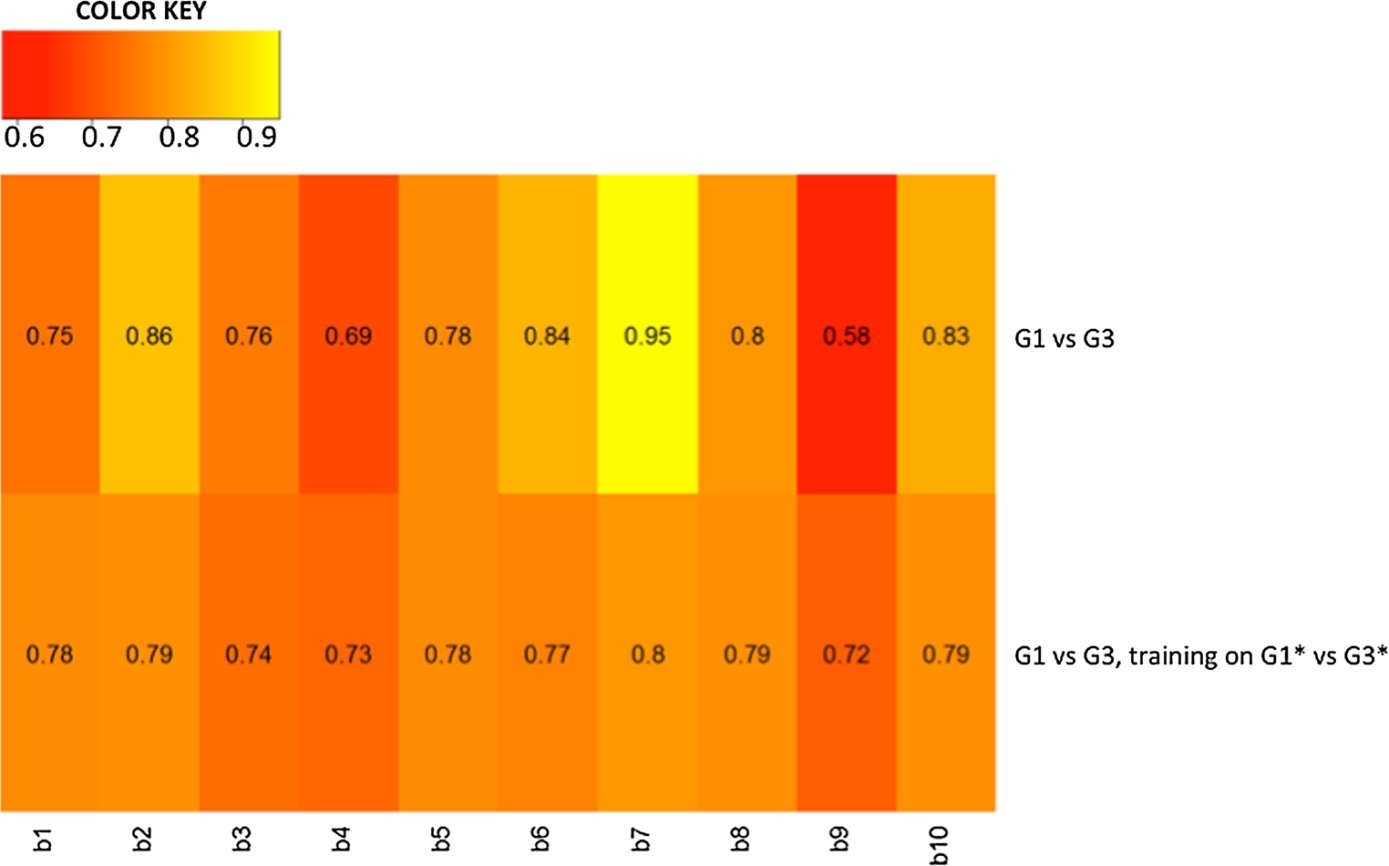
Heat maps depicting the predictive *performance* of *KPNA4* and *miR*-*567* classification for G1 versus G3 and for G1 versus G3, training on G1*–G3*, and testing on G1–G3 for each bootstrap b

Figure 7 shows heat maps of classification performances for G1 versus G3 BC samples, and for the re-classification G2 samples, training on G1*–G3*and testing on G1–G3 for each bootstrap. Mean AUC values showed a good performance of 0.78 [(CI 95%) 0.71–0.84] and 0.76 [(CI 95%) 0.74–0.77], respectively. The best performance in G1 versus G3 was obtained in the seventh bootstrap getting an AUC value 0.95. In the re-classification the best performance was 0.8.

## Discussion

In this study, we explored the role of *miR*-*567* in BC. Our study was based on the results coming from three BC experimental models: cellular model, mouse model, and human samples.

We investigated the role of *miR*-*567* in G3 versus G1 BC cell lines, and *miR*-*567* was found clearly differentially expressed (Fig. 1a). To better clarify the role that the downregulation of *miR*-*567* has in higher-grade tumor behavior and the genes that are potentially target of *miR*-*567* regulation, we overexpressed *miR*-*567* construct in MDA-MB-231 cells (Fig. 3a–d). *miR*-*567* upregulation has a drastic effect on cell growth, as revealed by the decrease in cell proliferation (Fig. 4a). In addition, its upregulation strongly affects the migration capability of the cells, as revealed by wound-healing assay (Fig. 4b–c).

In our previous publication [5] by in silico analyses, we had suggested *KPNA4* as a possible target of *miR*-*567*. Several members of the KPN family are activated in solid tumors (i.e., [9]) and in particular in BC [21]. Kpna4, a nuclear protein with a role in nucleocytoplasmic trafficking, could be involved in signal-transduction pathways and cell-cycle control [33, 34]. In Fig. 1b, we showed that MDA-MB-231 cell line has an increased level of *KPNA4* consistently with to the downregulation of *miR*-*567* expression. *miR*-*567* upregulation in MDA-MB-231 cell line decreases the expression level of *KPNA4* both as mRNA and protein in a statistically significant way (Fig. 3c–d), and luciferase assay confirms a direct interaction between *miR*-*567* and *KPNA4* 3′ UTR (Fig. 3e). Looking at the results from ex vivo experiments, *KPNA4* expression levels measured by RT-PCR in G3 versus G1 BC human samples revealed that this target gene is overexpressed in G3 (Fig. 2b), as expected [5]. These results suggest that in BC, *KPNA4* upregulation, as effect of *miR*-*567* downregulation, could contribute to the aggressive phenotype of G3 BC tumors.

A similar anti-proliferative effect obtained by *miR*-*567*-transfected cell analysis in in vitro study was obtained in vivo. We analyzed the tumors generated by MDA-miR compared to those obtained by MDA-ctr. We observed that MDA-miR-generated tumors showed a lower growth rate that those obtained by MDA-ctr cells (Fig. 5). These data confirm that *miR*-*567* is also able in vivo to control the growth rate of highly aggressive BC cells.

The clinical significance of *miR*-*567* in BC has not been evaluated yet. In the present study, *miR*-*567* downregulation has been detected in poor prognosis G3 human samples compared to better prognosis G1 human samples, suggesting a correlation between *miR*-*567* expression and prognosis (Fig. 2). The same results have been obtained for KPNA4 expression level analysis in G3 versus G1 BC samples (Fig. 2). Also, in silico analysis showed the good performances of *miR*-*567* and *KPNA4* to distinguish G1 and G3, and in the re-classification of G2, using an independent dataset (Fig. 7).

No publication associates *miR*-*567* expression levels to BC, although two papers showed, in silico, a correlation between *miR*-*567* expression and colon cancer (CRC) [8, 30]. In CRC, *miR*-*567* has been proposed as a controller of *SMAD4* pathway. *SMAD4*, a major component of the transforming growth factor β (TGF-β) signaling pathway, has been found activated in metastatic BC [32]. TGF-β pathway is crucial for the invasion and migration of BC cell in surrounding tissue [23], and it is upregulated in 81% of the patients with aggressive cancer [7, 15]. It is possible that *miR*-*567* is modulated by TGF-β pathway also in BC and, in turn, regulates some components of this pathway, as SMAD family members.

**Fig. 8:**
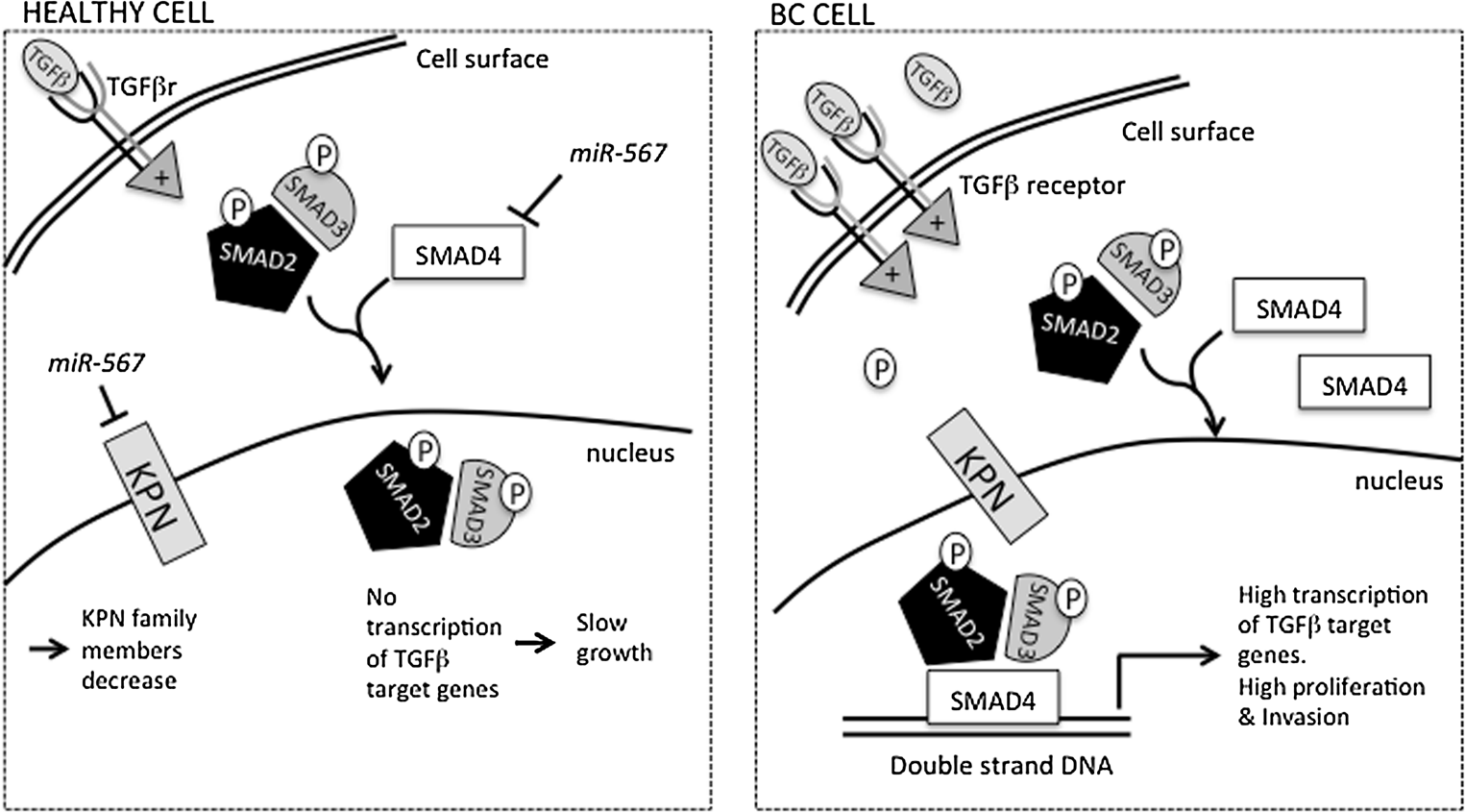
Hypothesis of *miR*-*567* modulation in healthy and BC cells. When *miR*-*567* is expressed, TGF-β, bound to its receptor (two subunits), activates the pathway that leads to the phosphorylation of downstream effectors (i.e., *SMAD2* and *3*) and to the downregulation of KPN family member. The last effect could be mediated by *miR*-*567* activity. *miR*-*567* could possibly also modulate *SMAD4* effector, needed for the transcriptional activation of TGF-β target genes. In aggressive BC cell, TGF-β is upregulated, and the higher activation of its pathway leads to transcription of genes involved in the proliferation control and invasion. The reduction of *miR*-*567* observed in aggressive BC could lead to increase of KPN family members, facilitating the growth of the tumor. TGFβr = TGFβ receptor

The higher expression of TGF-β is able to modulate a component of KPN karyopherin family, namely *KPNA2*, involved in the control of keratinocyte proliferation and differentiation [29]. We can hypothesize that in a normal cell, low levels of TGF-β allow the expression of *miR*-*567*, which in turn decrease KPN family members, regulating the cell growth. In highly aggressive BC, TGF-β is increased, causing possibly the strict decrease in *miR*-*567* expression and consequently the upregulation of *KPNA4,* increasing cell proliferation. This hypothesis is depicted in Fig. 8. However, further experiments are needed to confirm it.

In this study, we could propose *miR*-*567 as* a therapeutic tool: the use of *miR*-*567* mimic oligonucleotide could be effective in modulating BC growth through targeting of KPN family members. The balance of *miR*-*567* expression by mimic oligonucleotide administration could become a possible therapeutic strategy for poor prognosis BC. Several attempts have been made to use miRNAs as possible therapeutic tools [3], but further efforts are needed to achieve this purpose.

In conclusion, we found a high increase of *miR*-*567* in low-risk G1 versus G3 cell lines and even in the corresponding explanted tumors from xenografted mice. A significant difference has been also found between G1 and G3 human BC tissue samples. Our findings suggest that *miR*-*567* could act as an oncogene, as its downregulation decreases cell proliferation in poor prognosis BC, increasing expression level of *KPNA4*. *miR*-*567* might serve as a novel biomarker in the prognosis of BC and could be a regulator of *KPNA4* expression.
